# Portal Vein Thrombosis of a Newborn with Corrected Total Anomalous Pulmonary Venous Return

**DOI:** 10.4274/tjh.2013.0428

**Published:** 2015-08-01

**Authors:** Ufuk Çakır, Dilek Kahvecioğlu, Serdar Alan, Ömer Erdeve, Begüm Atasay, Tayfun Uçar, Saadet Arsan, Hasan Çakmaklı, Mehmet Ertem, Semra Atalay

**Affiliations:** 1 Ankara University Faculty of Medicine, Department of Pediatrics, Ankara, Turkey; 2 Ankara University Faculty of Medicine, Department of Pediatrics, Division of Neonatology, Ankara, Turkey; 3 Ankara University Faculty of Medicine, Department of Pediatrics, Division of Pediatric Cardiology, Ankara, Turkey; 4 Ankara University Faculty of Medicine, Department of Pediatrics, Division of Pediatric Hematology, Ankara, Turkey

**Keywords:** Total anomalous pulmonary venous return, Portal vein thrombosis, Anticoagulation therapy, Low-molecular-weight heparin

## Abstract

Total anomalous pulmonary venous return (TAPVR) is a rare and frequently isolated defect identified in 1% to 3% of all congenital heart diseases. To the best of our knowledge, portal vein thrombosis (PVT) associated with TAPVR has not been reported in the literature. We report a successfully managed PVT in a newborn with infracardiac-type TAPVR and review the literature. Anticoagulation therapies were used during the neonatal period to prevent thrombus progression. PVT should be kept in mind in TAPVR patients who have open heart repair with total correction. The treatment in each neonate should be individualized with consideration of the risk/benefit ratio.

## Abstract (tr)

Total anormal pulmoner venöz dönüş (TAPVD) anomalisi nadir ve çoğunlukla izole bir durumdur, bütün konjenital kalp hastalıklarının %1-3’ünü oluşturur. Bildiğimiz kadarıyla, TAPVD anomalisi ile ilişkili portal ven trombozu (PVT) literatürde daha önce yayınlanmamıştır. Burada başarı ile tedavi edilen infrakardiak tipte TAPVD anomalisine eşlik eden PVT’li bir yenidoğan olgusu literatür bilgileri ışığında sunulmaktadır. Tromboz progresyonunu engellemek için yenidoğan dönemi boyunca antikoagülan tedaviler uygulanmıştır. PVT, TAPVD anomalisi olan ve tam düzeltici açık kalp onarımı yapılan hastalarda akılda tutulmalıdır. Her bir yenidoğan için tedavi, risk ve yarar göz önünde tutularak bireyselleştirilmelidir.

## INTRODUCTION

Total anomalous pulmonary venous return (TAPVR) is an uncommon congenital cardiac anomaly with abnormal drainage of pulmonary venous blood into the systemic venous system. It may present as an isolated lesion or in combination with other cardiac defects. The clinical symptoms and signs are variable and depend on the associated cardiac anomaly. The prognosis of these patients is poor without surgical treatment. Prenatal diagnosis can offer more comprehensive perinatal management, reduce morbidity, and improve survival; however, TAPVR is rarely detected prenatally due to its low incidence and the difficulty of demonstrating the details of pulmonary venous anatomy with fetal echocardiography [1].

Postoperative systemic and pulmonary hypertension, pulmonary vein thrombosis, prolonged mechanical ventilation, heart failure, and mortality are reported complications after surgical correction of TAPVR in newborns [2,3,4]. To the best of our knowledge, portal vein thrombosis (PVT) associated with TAPVR has not been reported in the literature. We here report a successfully managed PVT in a newborn with infracardiac-type TAPVR and discuss the individualized management of this complication.

## CASE PRESENTATION

A 38-week male term infant of 2810 g was born to a 36-year-old mother by cesarean section. He was brought to the pediatric emergency room with respiratory distress, cyanosis, and apnea on the 14th day of life and was admitted to the neonatal intensive care unit (NICU). Physical examination on admission was unrevealing, except for tachypnea and cyanosis. His hemoglobin and platelet count on admission were 12.1 g/dL and 258x109/L, respectively. Normal chest X-ray and no response to hyperoxia testing pointed to a cardiac anomaly. Echocardiography revealed a dilated right atrium and ventricle and a small left atrium with infracardiac-type TAPVR (type 3), which was confirmed by computed tomography angiography. Informed consent was obtained.

The patient underwent a total surgical correction of cardiac surgery with cardiopulmonary bypass on the 19th postnatal day. Cardiac pumping for 120 min and hypothermia for 56 min were performed during the operation. The patient received dopamine, dobutamine, and milrinone infusions with furosemide for 2 days during the postoperative period, and he was extubated on the postoperative 3rd day. On the postoperative 6th day, his physical examination revealed abdominal distension and dilatation of superficial abdominal veins. Diagnostic portal Doppler ultrasonography (USG) demonstrated a 20x14 mm thrombus in the main portal vein with 4 mm of flow at the narrowest side.

Follow-up of the extension of thrombosis closely by Doppler USG under intravenous unfractionated heparin treatment was decided, with targeting of 50-80 s for activated partial thromboplastin time. The treatment decision was based on the location of the thrombus (main portal vein) and the probable risk of portal hypertension in the future. The choice of unfractionated heparin was made due to the recent postoperative period and the advantage of closer monitoring, in addition to easier management of complications such as bleeding. No extension or enlargement of the thrombus or any bleeding complications were observed in 2 consecutive days and the treatment was changed to subcutaneous low-molecular-weight heparin (LMWH) at a dose of 1.7 mg/kg twice a day [5]. Antifactor Xa level during the treatment ranged between 0.5 and 0.9 units/mL. Clinical symptoms such as abdominal distension and dilatation of superficial veins regressed in the following weeks and the patient was discharged on the postoperative 28th day. Protein C, protein S, and antithrombin III values were 59.4%, 94.9%, and 90.4%, respectively, during screening for thrombophilia. The patient was also evaluated for hereditary thrombophilic mutations and was found to be heterozygous for methylenetetrahydrofolate reductase mutation. His homocysteine level was 17.03 µmol/L and 14 µmol/L (normal range: 5-14 µmol/L) on the 28th and 44th days, respectively. Factor V Leiden and prothrombin 20210Aa mutations were not detected. LMWH was discontinued after the demonstration of complete resolution of the PVT by USG at the 8th week of treatment. For follow-up, he was evaluated at 6 months of age with no signs of PVT or portal hypertension.

## DISCUSSION AND REVIEW OF THE LITERATURE

TAPVR is a rare and frequently isolated defect identified in 1% to 3% of all congenital heart diseases. In TAPVR systemic and pulmonary veins return to the right heart circulation, creating an obligate right-to-left atrial shunt to provide left ventricular preload [4]. Open heart repair with total correction, such as in our patient, is necessary in most cases. One of the most important complications of open heart repair with total correction is thrombosis, especially in pulmonary veins. Our patient developed PVT, which has not been reported previously.

Neonatal PVT has been described as a rare event, but it is becoming more commonly recognized. Estimates range from 1 in 100,000 live births to 1 to 36 per 1000 NICU admissions [6]. The majority of cases remain unrecognized in the neonatal period and manifest later in childhood [5]. PVT is the major cause of extrahepatic portal hypertension and gastrointestinal bleeding in children, and the portal hypertension is most commonly the result of an organized thrombus in the portal vein [2].

The etiology of PVT is different in neonates from that in children and adults. In adults, it is most frequently secondary to cirrhosis, whereas, in older children, PVT is related to liver transplantation, intraabdominal sepsis, splenectomy, sickle cell anemia, and antiphospholipid antibodies [5,7]. In addition, PVT secondary to pylephlebitis due to urinary tract infection was presented in an adult case [8]. PVT in neonates commonly occurs secondary to the placement of an umbilical vein catheterization (UVC) with or without infection. In a large retrospective review of 133 infants identified with PVT, an umbilical catheter was inserted in 97 (73%) and in half of them the catheter was misplaced at an intrahepatic location where venous flow rate was low. Our patient did not have a UVC, infection, or meaningful hereditary thrombophilia.

We thought that congenital vascular malformation, hypothermia, and cardiac pump could be possible causes of the thrombosis in the presented case. Poor outcome was reported in 36 infants with intrahepatic lobe atrophy (30 cases) or portal hypertension (6 cases) [6]. In our case, early spontaneous resolution occurred. Scarce presenting clinical signs could be the reason why PVT is a relatively rare diagnosis in the NICU setting [6,9,10,11]. We think that the incidence of PVT in postoperative cardiac patients is not exactly known and we suggest that these patients should be followed for this possible complication.

Although the clinical and laboratory signs are not prominent in neonates with PVT, dilation of abdominal superficial vessels in addition to abdominal distension may be an alarming physical finding.

Thrombocytopenia can be seen at the time of diagnosis in PVT cases, but it is not specific for PVT. Consumption of platelets secondary to sepsis and disseminated intravascular coagulation may explain the low platelet count in the acute stage. In the late chronic stage, a low platelet count may be most likely secondary to hypersplenism with portal hypertension [5]. In a series by Morag et al., thrombocytopenia was reported in 26/133 infants (19.5%); in 13 of these infants, it was secondary to other conditions, including necrotizing enterocolitis or sepsis [6]. There can be mild liver dysfunction in children with PVT. The extent to which similar abnormalities occur in neonates is less clear. In a series of neonatal PVT cases, 9 out of 133 (7%) neonates had abnormal liver enzymes as an indication for ultrasound, which subsequently identified the presence of PVT [6]. Our patient did not demonstrate thrombocytopenia or elevated liver enzymes at the time of diagnosis or during his hospital stay. Prognosis of PVT is associated with ultrasound grading and grade 3 PVT may lead to lobe atrophy and portal hypertension.

Although there are insufficient data to make strong recommendations regarding anticoagulation therapy for neonatal PVT, the options include conventional anticoagulation therapy, short-term anticoagulation therapy, or close monitoring of the thrombus with objective tests and the use of anticoagulation therapy if thrombus extension occurs [5]. The treatment in each neonate should be individualized with consideration of the risk/benefit ratio [5]. We preferred anticoagulation therapy with unfractionated heparin and followed the patient closely for a possible extension during the first 2 days. After the second USG, which did not demonstrate any extension, we changed the treatment to LMWH for the ease of monitoring. Thrombolytic therapy is usually reserved for major vessel occlusion such as inferior vena cava, renal veins, or right atrium with organ compromise, and it is contraindicated in patients who underwent general surgery within the last 10 days [5].

In conclusion, PVT should be kept in mind in TAPVR cases with open heart repair with total correction. The treatment in each neonate should be individualized with consideration of the risk/benefit ratio. Patients should be followed closely and thrombolytic therapy should be reserved for major vessel occlusion with organ compromise. Anticoagulation therapy with unfractionated heparin and LMWH may be used during the neonatal period to prevent future complications. Those patients with PVT should be followed for the risk of hypertension.
